# Effect of low-dose aspirin intervention on pre-eclampsia prevention in high-risk pregnant women and its impact on postpartum hemorrhage

**DOI:** 10.3389/fmed.2024.1414697

**Published:** 2024-10-25

**Authors:** Fangfang Zhang, Huijuan Wang

**Affiliations:** Department of Obstetrics and Gynecology, Xi’an People's Hospital (Xi’an Fourth Hospital), Xi’an, Shaanxi, China

**Keywords:** pre-eclampsia, high-risk pregnancy, low-dose aspirin, intervention, postpartum hemorrhage

## Abstract

**Background:**

Pre-eclampsia, characterized by hypertension and organ dysfunction during pregnancy, poses significant risks to both maternal and fetal health. Aspirin, known for its antiplatelet properties, has been extensively utilized to mitigate pregnancy-related complications. However, the efficacy of low-dose aspirin in managing pre-eclampsia among high-risk pregnant women and its potential impact on postpartum hemorrhage remain contentious topics.

**Methods:**

A retrospective analysis was conducted on 344 pregnant women diagnosed with high-risk factors for pre-eclampsia. Among them, 152 received intervention with low-dose aspirin, while the rest did not receive it. The incidence of pre-eclampsia, as well as related complications and outcomes associated with bleeding, were compared and evaluated between the two groups.

**Results:**

The study findings indicate a significant reduction in the incidence of pre-eclampsia among pregnant women receiving low-dose aspirin intervention, along with a significantly reduced risk of complications. Additionally, there was no significant statistical difference in postpartum hemorrhage between the two groups (*p* > 0.05). The safety profile of aspirin usage was found to be favorable.

**Conclusion:**

Low-dose aspirin demonstrates promising efficacy as an intervention strategy for high-risk preeclamptic women. It does not increase the risk of postpartum hemorrhage and reduces the occurrence of complications associated with preeclampsia. Therefore, low-dose aspirin presents a potential preventive measure against adverse outcomes associated with high-risk pregnancies related to preeclampsia. Further research is necessary to validate and elucidate the optimal dosage and timing of administration for maximal benefits.

## Introduction

Pre-eclampsia is a multisystem disorder characterized by hypertension and organ dysfunction, posing significant risks to the health of pregnant women and their fetuses. Globally, it remains a leading cause of morbidity and mortality among pregnant and perinatal women, especially in low-and middle-income countries where access to antenatal care may be limited. Despite advances in obstetric care, the precise etiology of pre-eclampsia remains elusive, complicating efforts to devise effective prevention and management strategies (1–3).

Aspirin, a well-established antiplatelet agent, has garnered attention for its potential role in mitigating pregnancy-related complications, including pre-eclampsia (4, 5). Low-dose aspirin therapy has been extensively studied as a preventive measure due to its favorable safety profile and plausible mechanisms of action such as reducing inflammation and improving placental perfusion. However, the impact of low-dose aspirin intervention on pregnant women at high risk of developing PE and outcomes such as postpartum hemorrhage remains a subject of debate and ongoing research. Studies by Nzelu et al. (6) through a cost-effectiveness cohort study found clinical benefits and economic savings with pre-pregnancy screening and targeted aspirin prophylaxis, while a meta-analysis by D’Antonio suggested a reduction in pre-eclampsia risk in women with twin pregnancies taking aspirin (7). However, the conclusion of Richards’ systematic review suggests that there is insufficient evidence to demonstrate a significant change in the likelihood of developing superimposed pre-eclampsia, preterm birth, or perinatal mortality rates among women with chronic hypertension using low-dose aspirin. However, it is still emphasized that randomized controlled trials are needed to elucidate the actual role of aspirin in influencing outcomes in pregnant women with twin pregnancies and perinatal outcomes (8, 9).

This study aims to contribute to the existing literature by examining the impact of low-dose aspirin intervention on pregnant women at high risk of developing PE. Through a retrospective analysis of a considerable group, we seek to assess the association between the use of low-dose aspirin and the incidence of postpartum hemorrhage and other relevant outcomes. Findings from this study could have implications for clinical practice, potentially providing guidance and interventions to improve maternal and fetal health outcomes.

Therefore, understanding the efficacy and safety of low-dose aspirin is crucial in this context, offering the potential to enhance antenatal care and global maternal health. By elucidating the role of low-dose aspirin in managing pre-eclampsia and its sequelae, this study endeavors to contribute to ongoing efforts to improve pregnancy outcomes and alleviate the global burden of morbidity and mortality among pregnant and perinatal women.

## Materials and methods

This retrospective group study aimed to investigate the impact of low-dose aspirin intervention on pregnant women at high risk of developing PE and its association with postpartum hemorrhage. The study was conducted between June 2021 and December 2023 at Xi’an People’s Hospital. This retrospective study was approved by the Ethics Committee of Xi’an People’s Hospital and followed the Declaration of Helsinki. All patients provided informed consent.

Inclusion criteria:

1. Women aged 18–55 years old.
2. First ongoing pregnancy beyond the first trimester. This includes women whose previous pregnancies ended before reaching the second trimester (e.g., early miscarriages).
3. Fetal age between 12 and 20 weeks with a viable fetus at the time of aspirin administration.
4. High-risk definition for developing preeclampsia:

- At least one high-risk factor, including diabetes (type 1 or 2), or chronic hypertension; or
- At least two intermediate-risk factors, including obesity, advanced maternal age (35 years or older), family history of preeclampsia (mother and/or sister), or nulliparity.

Exclusion criteria:

1. Allergy to aspirin.
2. Respiratory system allergy.
3. Lack of tolerance to the study due to severe cardiac, hepatic, or renal diseases.
4. Autoimmune diseases.
5. Mental illness.
6. History of alcohol or drug abuse within the past 6 months.
7. Severe missing information.
8. Previous participation in drug trials.

The term “first pregnancy” in this study refers to the first ongoing pregnancy beyond the first trimester, including pregnancies that did not result in live births previously. Women with previous pregnancies that ended in early miscarriage were considered nulliparous for this study. Potential previous miscarriages were taken into account, and women who had experienced early pregnancy losses were included under the criteria of nulliparity if they had not carried a pregnancy to a viable stage before. The criterion “history of preeclampsia” was divided into personal history (previous pregnancies with preeclampsia) and family history (mother or sister with preeclampsia).

In the group, 152 pregnant women received low-dose aspirin intervention, while the remainder did not receive any aspirin treatment. The low-dose aspirin intervention involved administering aspirin at a dose of 150 mg/day (10), initiated between 12 and 20 weeks of gestation for women identified as high-risk for developing pre-eclampsia, and continued until 36 weeks of gestation. The decision to administer aspirin was made based on clinical guidelines and individual risk assessments by a consistent team of experienced obstetricians. The timing of medication administration is based on existing research evidence, indicating that this period is critical for placental development. Administering the medication during this phase is more effective in improving placental blood flow and reducing inflammation. The decision to discontinue the medication at 36 weeks is also based on existing research evidence. Stopping the medication at 36 weeks aims to continue protecting maternal and fetal safety while minimizing the risk of bleeding. Variations in practice patterns and patient-specific factors influenced whether aspirin was prescribed, leading to the observed distribution of treatment.

All patients who received low-dose aspirin were instructed to discontinue the medication at 36 weeks of gestation. This timing was chosen to balance the benefits of pre-eclampsia prevention with the need to minimize the risk of post-partum hemorrhage.

### Preeclampsia

The diagnosis of preeclampsia was based on the criteria set forth by the American College of Obstetricians and Gynecologists (ACOG), which include (11):

1. Systolic blood pressure ≥ 140 mmHg or diastolic blood pressure ≥ 90 mmHg after 20 weeks of gestation in a previously normotensive woman.
2. New onset proteinuria ≥300 mg in a 24-h urine collection or a urine protein-to-creatinine ratio ≥ 0.3 mg/dL.

In women with pre-pregnancy hypertension, PE was diagnosed if there was new onset of one or more of the following after 20 weeks of gestation:

1. Proteinuria ≥300 mg in a 24-h urine collection or a urine protein-to-creatinine ratio ≥ 0.3 mg/dL.
2. Thrombocytopenia: platelet count <100,000/mL.
3. Renal insufficiency: serum creatinine >1.1 mg/dL or a doubling of serum creatinine in the absence of other renal disease.
4. Impaired liver function: elevated blood concentrations of liver transaminases to twice normal concentration.
5. Pulmonary edema.
6. New-onset headache unresponsive to medication and not accounted for by alternative diagnoses or visual symptoms.

### Postpartum hemorrhage

The criteria for postpartum hemorrhage were defined as (12):

1. Blood loss ≥500 mL after vaginal delivery or ≥ 1,000 mL after cesarean delivery.
2. Clinical signs of hypovolemic shock, such as a drop in hematocrit, tachycardia, or hypotension.

Data on patient demographics, medical history, antenatal care, obstetric outcomes, and aspirin treatment were extracted from Electronic Medical Record (EMR) and obstetric databases. Relevant variables included obesity status, advanced maternal age, chronic hypertension, diabetes mellitus, history of pre-eclampsia, gestational age at enrollment, and occurrence of postpartum hemorrhage. Data collection was performed by trained medical personnel and cross-checked to ensure accuracy and completeness.

Statistical analysis was conducted using appropriate software (e.g., SPSS [version 26.0], R). Descriptive statistics were used to summarize patient characteristics and outcomes. Continuous variables were expressed as mean ± standard deviation (SD) or median [interquartile range (IQR)], while categorical variables were presented as frequencies and percentages. Comparison analysis between the intervention and control groups was performed using chi-square test, Student’s *t*-test, or Mann–Whitney U test. Univariate and multi variate logistic regression analyses were used to identify risk factors influencing pre-eclampsia-related complications and postpartum hemorrhage. The selection criteria for the regression analyses in Table 1 included established high-risk factors for pre-eclampsia (PE) and their potential impact on the outcomes of interest. These factors were chosen to quantify their specific contributions and interactions in our study population. A *p* value <0.05 was considered statistically significant.

**Table 1:** Univariable and multivariable logistic regression analysis for pre-eclampsia patients in the entire cohort.

	Univariate analysis			Multivariate analysis		
	*p*	HR	95% confidence interval	*p*	HR	95% confidence interval
Obese (pre-BMI ≥ 28 kg/m^2^)	**0.011**	1.285	1.044–1.467	**0.023**	1.311	1.158–1.621
Yes/No						
Age	**0.001**	1.379	1.102–1.564	**0.012**	1.288	1.049–1.482
> 35 years/≤ 35 years						
Chronic hypertension	**0.006**	1.410	1.267–1.772	**0.002**	1.522	1.378–1.908
Yes/No						
Pre-existing diabetes	0.315	1.776	0.716–2.531			
Yes/No						
History of pre-eclampsia	**<0.001**	2.134	1.378–2.931	**<0.001**	1.982	1.466–2.678
Yes/No						
Nulliparity	0.388	1.166	0.697–1.455			
Yes/No						
Gestational age at enrollment (weeks)	0.512	1.821	0.688–3.781			
> 16/≤ 16						
Aspirin use	**<0.001**	0.782	0.561–0.912	**<0.001**	0.695	0.422–0.913
Yes/No						

BMI, Body mass index. Bold means there is a statistical difference.

## Results

**Figure 1:**
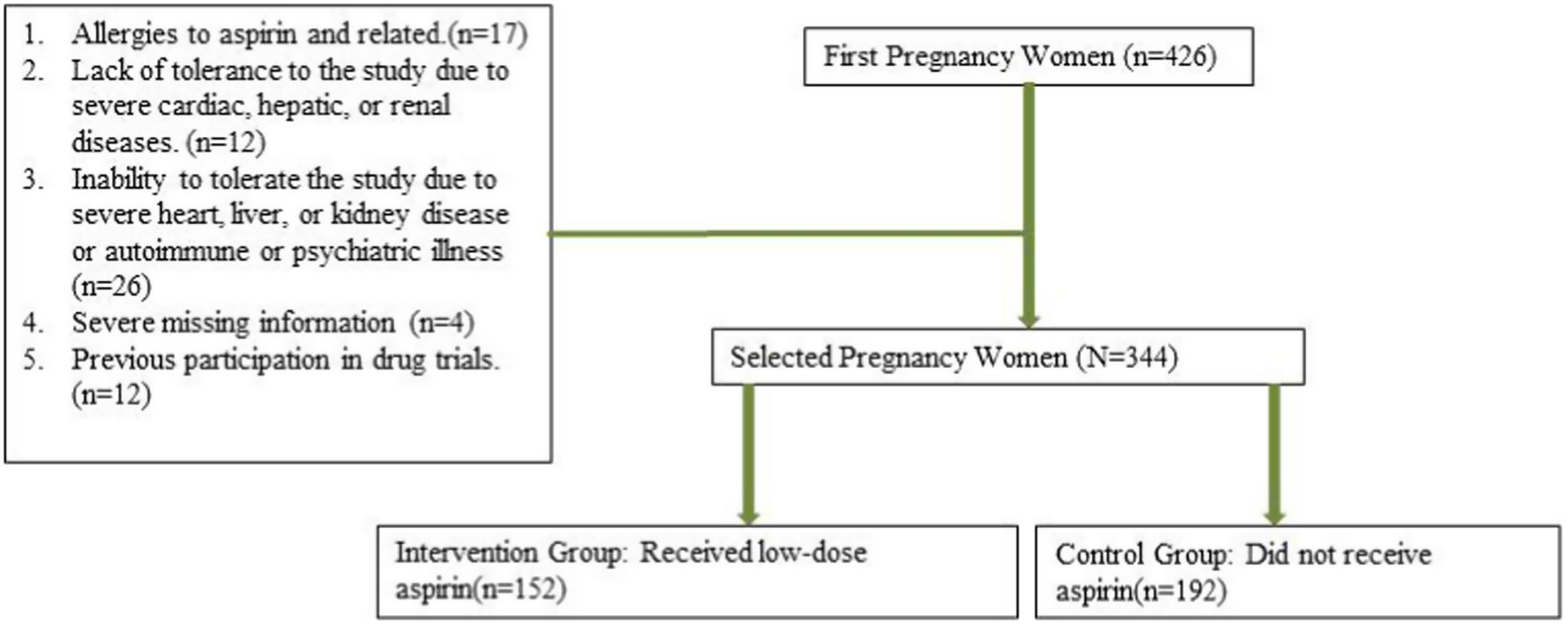
Patients flowchart.

A total of 344 patients at high risk of developing PE were enrolled in the study, among whom 152 patients received aspirin treatment (Figure 1). The incidence of pre-eclampsia was significantly lower in the aspirin group (13.2%) compared to the non-aspirin group (22.4%) (*p* = 0.035). The incidence of postpartum hemorrhage did not significantly differ between the aspirin group (5.9%) and the non-aspirin group (6.8%) (*p* = 0.827). The aspirin group had a significantly lower incidence of placental abruption (5.9%) compared to the non-aspirin group (15.6%) (*p* = 0.006). The distribution of other variables did not show statistically significant differences between the groups (Table 2).

**Table 2:** Baseline characteristics of high-risk pregnant women, with or without aspirin use (*n* = 344).

		Aspirin group (*N* = 152)	No-Aspirin group (*N* = 192)	*p* value
	
Obese (pre-BMI ≥28 kg/m^2^) (%)				0.808
	Yes	40 (26.3)	53 (27.6)	
	No	112 (73.7)	139 (72.4)	
Age (%)				0.936
	18–25 years	30 (19.7)	34 (17.7)	
	25–35 years	64 (42.1)	87 (45.3)	
	35–45 years	50 (32.9)	61 (31.8)	
	> 45 years	8 (5.3)	10 (5.2)	
Chronic hypertension (%)				0.277
	Yes	77 (50.7)	85 (44.3)	
	No	75 (49.3)	107 (55.7)	
Pre-existing diabetes (%)				0.802
	Yes	36 (23.7)	48 (25.0)	
	No	116 (76.3)	144 (75.0)	
History of pre-eclampsia				0.733
	Family history	41 (27.0)	48 (25.0)	
	Personal history	12 (7.9)	12 (6.3)	
	No	99 (65.1)	132 (68.8)	
Nulliparity				0.821
	Yes	52 (34.2)	68 (35.4)	
	No	100 (65.8)	124 (64.6)	
Gestational age at aspirin administration (weeks)				0.211
	>16	47 (30.9)	72 (37.5)	
	≤16	105 (69.1)	120 (62.5)	
Pre-eclampsia				0.035
	Yes	20 (13.2)	43 (22.4)	
	No	132 (86.8)	149 (77.6)	
Postpartum hemorrhage				0.827
	Yes	9 (5.9)	13 (6.8)	
	No	143 (94.1)	179 (93.2)	

BMI, Body mass index.

Univariate analysis identified obesity (OR = 1.285, *p* = 0.011), age (OR = 1.379, *p* = 0.001), chronic hypertension (OR = 1.410, *p* = 0.006), history of pre-eclampsia (OR = 2.134), and aspirin use (OR = 0.782, *p* < 0.001) as significant predictors for pre-eclampsia. Multivariate analysis confirmed aspirin use (OR = 0.782, *p* < 0.001) as an important protective factor (Table 1).

Univariate analysis showed that obesity (OR = 1.304, *p* = 0.021), age (OR = 1.247, *p* = 0.002), chronic hypertension (OR = 1.315, *p* = 0.012), pre-existing diabetes (OR = 1.820, *p* = 0.001), and history of pre-eclampsia (OR = 2.881, *p* < 0.001) were significant predictors for postpartum hemorrhage. Multivariate analysis confirmed age (OR = 1.314, *p* = 0.013), pre-existing diabetes (OR = 1.720, *p* = 0.020), and history of pre-eclampsia (OR = 2.053, *p* = 0.034) as significant factors. Aspirin use was not a significant risk factor for postpartum hemorrhage (*p* = 0.220) (Table 3).

**Table 3:** Univariable and multivariable logistic regression analysis of postpartum hemorrhage in the entire cohort.

	Univariate analysis			Multivariate analysis		
	*p*	HR	95% confidence interval	*p*	HR	95% confidence interval
Obese (pre-BMI ≥ 28 kg/m^2^)	0.021	1.304	1.078–1.388	0.136	1.288	1.078–1.388
Yes/No						
Age	0.002	1.247	1.069–1.478	0.013	1.314	1.187–1.684
> 35 years/≤ 35 years						
Chronic hypertension	0.012	1.315	1.127–1.671	0.255	1.408	0.843–1.913
Yes/No						
Pre-existing diabetes	0.001	1.820	1.364–2.788	0.020	1.720	1.246–2.354
Yes/No						
History of pre-eclampsia	<0.001	2.881	2.092–4.361	0.034	2.053	1.532–2.613
Yes/No						
Nulliparity	0.234	1.008	0.755–1.284			
Yes/No						
Gestational age at enrollment (weeks)	0.410	1.018	0.790–1.277			
> 16 /≤ 16						
Aspirin use	0.220	1.355	0.726–1.613			
Yes/No						

BMI, Body mass index.

Table 4 shows the incidence of complications based on aspirin usage. Placental abruption was significantly lower in the aspirin group (*p* = 0.006), while the incidence of gestational hypertension and other related complications did not differ significantly between the two groups (Table 4).

**Table 4:** Delivery outcomes and complications in high-risk pregnant women with or without aspirin use (*n* = 344).

		Aspirin group (*N* = 152)	No-Aspirin group (*N* = 192)	*p* value

Preterm delivery at <37 gestational weeks (%)				0.024
	Yes	6 (3.9)	20 (10.4)	
	No	146 (96.1)	172 (89.6)	
Gestation hypertension (%)				0.835
	Yes	10 (6.6)	15 (7.8)	
	No	142 (93.4)	177 (92.2)	
HELLP syndrome (%)				1.000
	Yes	0 (0.0)	0 (0.0)	
	No	152 (100.0)	192 (100.0)	
Placental abruption (%)				0.027
	Yes	1 (0.7)	9 (4.7)	
	No	149 (99.3)	183 (95.3)	
Premature rupture of membranes (%)				0.286
	Yes	5 (3.3)	11 (5.7)	
	No	147 (96.7)	181 (94.3)	
Placental implantation spectrum disorders (%)				0.401
	Yes	2 (1.3)	5 (2.6)	
	No	150 (98.7)	187 (97.4)	
Other complications (%)				0.018
	Yes	5 (3.3)	18 (9.9)	
	No	147 (96.7)	164 (90.1)	

HELLP, Hemolysis, elevated liver enzymes, and low platelets.

The study group includes some nulliparous patients, but not all. This distinction is shown in data on complications like Preterm Delivery, Premature Rupture of Membranes, and Placental Implantation Spectrum Disorders.

## Discussion

Pre-eclampsia (PE) is an obstetric condition affecting 3–8% of pregnant women, posing a significant concern in clinical and obstetric care due to its potential for severe complications affecting both maternal and fetal health, impacting patient prognosis, and increasing hospitalization costs (13, 14). This retrospective study examines the impact of low-dose aspirin intervention on pregnant women at high risk of developing PE and its association with related complications, including postpartum hemorrhage. The findings offer crucial insights into how low-dose aspirin affects patient outcomes and its significance in clinical practice.

The baseline characteristics of the study population highlight significant demographic and clinical features of pregnant women at high risk of developing PE. Common risk factors such as advanced maternal age, chronic hypertension, and obesity were prevalent among participants. These findings underscore the importance of early identification of high-risk individuals to implement appropriate interventions and mitigate adverse outcomes. Bartsch et al. (15). published a meta-analysis identifying several practical clinical risk factors that may determine “high-risk” status for PE in early pregnancy, including nulliparity, advanced maternal age, high body mass index, chronic hypertension, pre-existing diabetes (type 1 or type 2), chronic kidney disease, systemic lupus erythematosus, antiphospholipid antibody syndrome, assisted reproduction, and multiple gestations.

Aspirin is currently the recommended sole medication for preventing PE (16, 17). Since its use began in 1979, with daily doses ranging from 50 to 150 mg for women of different gestational ages, both low-risk and high-risk, our study suggests that low-dose aspirin intervention may reduce the incidence of PE in high-risk pregnant women. Significantly fewer patients in the aspirin-treated group developed PE compared to the non-aspirin-treated group, emphasizing the potential preventive effect of aspirin in this population. These results align with previous research indicating the efficacy of aspirin in reducing PE risk. Ma’ayeh et al. (18). suggest using low-dose aspirin as recommended by professional associations to prevent PE in high-risk women. Some experts even advocate for universal aspirin use during pregnancy, given its extensive study for safety and potential to alleviate burden of PE, improve maternal and fetal outcomes, and reduce medical costs.

The observed rates of pregnancy-related complications such as gestational hypertension, HELLP syndrome, and placental abruption further elucidate the clinical outcomes associated with aspirin use (19–21). While the incidence of placental abruption was significantly lower in the aspirin group, other complications did not show significant differences, suggesting a nuanced relationship between aspirin intervention and specific pregnancy-related complications, warranting further research into potential mechanisms.

Different studies offer varying recommendations on the timing of aspirin administration. For example, a randomized controlled trial by Ebrashy advocates starting aspirin as early as 14 weeks of gestation (22), while a meta-analysis by Roberge et al. (23) suggests beginning at 16 weeks, with little impact on the risks of PE, severe PE, and fetal growth restriction when started at this time. In our study, aspirin administration was initiated between 12 and 20 weeks to align with practical patient management, ensuring early identification and treatment of high-risk women for optimal maternal and fetal outcomes.

This study contributes to the ongoing discussion on the use of aspirin in treating PE and its consequences. While previous studies suggest potential benefits of aspirin in reducing PE risk, our findings underscore the complexity of the issue (24–26). Further randomized controlled trials are necessary to clarify the role of aspirin in influencing postpartum outcomes in high-risk women. The effectiveness of our risk factor criteria is demonstrated by the significantly higher prevalence of PE (18%) in our study group compared to the general population, consistent with other high-risk PE groups reporting prevalence rates ranging from 15 to 25% (27–29).

In conclusion, this study provides valuable insights into the role of low-dose aspirin intervention in pregnant women at high risk of developing PE. The findings suggest potential benefits in reducing certain complications, such as placental abruption, though further research is needed to clarify aspirin’s overall impact on maternal and fetal outcomes. By enhancing our understanding of aspirin’s efficacy and safety in this population, future research can offer robust evidence for clinical practice and improve outcomes for women at high risk of developing PE.

## Data Availability

The raw data supporting the conclusions of this article will be made available by the authors, without undue reservation.
